# Efficacy of Conventional and Organic Insecticides against *Scaphoideus titanus*: Field and Semi-Field Trials

**DOI:** 10.3390/insects14020101

**Published:** 2023-01-17

**Authors:** Stefan Cristian Prazaru, Lisa D’Ambrogio, Martina Dal Cero, Mirko Rasera, Giovanni Cenedese, Enea Guerrieri, Marika Pavasini, Nicola Mori, Francesco Pavan, Carlo Duso

**Affiliations:** 1 Department of Agronomy, Food, Natural Resources, Animals and Environment, University of Padova, Viale dell’Università 16, 35020 Legnaro, Italy; 2 Department of Biotechnology, Verona University, 37134 Verona, Italy; 3 Department of Agricultural, Food, Environmental and Animal Sciences, University of Udine, Via delle Scienze 206, 33100 Udine, Italy

**Keywords:** phytoplasma vector, leafhoppers, insecticides, residual effects, Integrated Pest Management, Flavescence dorée

## Abstract

**Simple Summary:**

The efficacy of conventional and organic insecticides on nymphs and adults of *Scaphoideus titanus*, the main vector of phytoplasmas associated with the grapevine yellow Flavescence dorée, was evaluated. In trials carried out in the open field, etofenprox and deltamethrin proved to be the best conventional insecticides, while pyrethrins were the most impactful among organic insecticides. Insecticide residual effects were evaluated in semi-field and field conditions. Acrinathrin showed the most significant residual effects, followed by other pyrethroids. Organic insecticides gave poor results in terms of residual efficacy. In field trials there was a loss of residual activity coinciding with higher temperatures. Implications for *S. titanus* control strategies are discussed.

**Abstract:**

*Scaphoideus titanus* is the main vector of phytoplasmas associated with Flavescence dorée (FD), one of the most serious threats to viticulture in many European countries. To minimize the spread of this disease, mandatory control measures against *S. titanus* were decided in Europe. In the 1990s, the repeated application of insecticides (mainly organophosphates) proved to be an effective measure to control the vector and the related disease in north-eastern Italy. These insecticides and most of the neonicotinoids were recently banned from European viticulture. Serious FD issues detected in the recent years in northern Italy could be related to the use of less effective insecticides. Trials aimed at evaluating the efficacy of the most used conventional and organic insecticides in the control of *S. titanus* have been performed in semi-field and field conditions to test this hypothesis. In efficacy trials, carried out in four vineyards, etofenprox and deltamethrin proved to be the best conventional insecticides, while pyrethrins were the most impactful among organic insecticides. Insecticide residual activity was evaluated in semi-field and field conditions. Acrinathrin showed the most significant residual effects in both conditions. In semi-field trials, most of the pyrethroids were associated with good results in terms of residual activity. However, these effects declined in field conditions, probably due to high temperatures. Organic insecticides showed poor results in terms of residual efficacy. Implications of these results in the context of Integrated Pest Management in conventional and organic viticulture are discussed.

## 1. Introduction

The leafhopper *Scaphoideus titanus* Ball is the main vector of Grapevine flavescence dorée phytoplasma (FDP) [1,2], belonging to the elm yellows group (16SrV) [3]. Flavescence dorée (FD) is considered one of the most important diseases in European vineyards, and is currently causing serious damage to grapevine yield and quality in many European regions [4,5,6]. Originating from North America, *S. titanus* was detected first in France [7] then in Italy [8]. In the following years, *S. titanus* spread to many other European countries, from Portugal to Romania [9]. More recently, it has been detected in the north Caucasus [10]. Monophagous on grapevines, *S. titanus* can acquire phytoplasmas as nymphs by feeding on infected plants. The latency period is about 4–5 weeks, and the infectious adults can transmit phytoplasmas to healthy plants for the rest of their life even across large areas [9,11]. Moreover, *S. titanus* can acquire the phytoplasma as an adult, becoming infectious within 1-2 weeks [12]. *Scaphoideus titanus* develops one generation per year and overwinters as eggs. Egg hatching starts in May and lasts for about two months [13]. Females with mature eggs can be found from July (when oviposition starts) until late October [8,14,15]. Adult females live an average of 60 days, some of them 70–100 days, while adult male longevity is shorter [15]. The short latency period combined with the long survival implies that adults have a long inoculation period.

FDP is a quarantine pathogen, and its control is regulated by mandatory measures including the rouging of infected grapevines and the chemical control of *S. titanus* [4]. An area-wide and prompt monitoring of the vector is the pre-requisite to design a rational control strategy. Moreover, the vector sampling is a key action for pest management as it allows us to identify the best timing for insecticide applications. With a high density of *S. titanus*, two insecticide applications are required: the first one against third instar nymphs (before they become infectious) and the second one after two weeks, to suppress newly hatched nymphs [16]. Chemical control must be integrated with cultural measures, such as the removal of branches from winter pruning and suckers. The elimination of pruning remains reduces the stock of eggs in the vineyard [17], while the removal of suckers growing along the vertical trunk could contribute to decrease *S. titanus* population levels, frequently higher on suckers than within the canopy [18,19]. The presence of abandoned vineyards and American vines growing spontaneously in woodland can represent a source of infectious vectors for neighboring vineyards [20,21,22], and should be removed before the appearance of adults which can fly up to 300 m [23].

One of the most severe FD outbreaks in Europe took place in the Veneto region, north-eastern Italy, in the 1990s [24]. Chemical control measures against *S. titanus* gave satisfactory effects in controlling the spread of the disease [16,21,25]. Insecticides used for this purpose (e.g., organophosphates, some chitin-inhibitors) were characterized by a long residual effect often associated with contact activity. The most used active ingredients were fenitrothion, chlorpyriphos-ethyl, chlorpyrifos-methyl, buprofezin and flufenoxuron [26]. More recently, the neonicotinoid thiamethoxam was largely employed with satisfactory results [27,28,29]. In recent years, all these active ingredients have been removed from the European market (in particular, chlorpyrifos-ethyl, chlorpyrifos-methyl and thiamethoxam were applied until 2019) because of concerns for human health and the environmental impact. At the same time, other insecticides such as the pyrethroids etofenprox and lambda-cyhalothrin were classified as candidates for substitution by the European Commission (Regulation (EC) No 1107/2009) [30], and their use in the IPM guidelines developed in many Italian regions was restricted. In the last three years, the main active ingredients considered in IPM guidelines in Italy for the control of *S. titanus* have been acetamiprid, flupyradifurone and tau-fluvalinate. In this context, issues with FD increased in northern Italy, and changes in the active ingredients used in vineyards were suspected to be involved in this event. 

In Italian organic viticulture, few active ingredients are allowed for the control of *S. titanus.* Pyrethrins are commonly used, but their low persistence makes the execution of repeated applications necessary [16,31,32,33]. Their effects may be enhanced when used in combination with adjuvants. The effectiveness of other products based on azadirachtin, potassium salts of fatty acids or *Beauveria bassiana* is considered to be from moderate to low [32,34]. It is not surprising that FD is a key problem for organic farms.

In this work, we evaluated the effectiveness of insecticides authorized in Italy for the control of *S. titanus* in conventional and organic viticulture. Specific trials, both in semi-field and field conditions, were performed to evaluate the efficacy and the residual activity of the insecticides on nymphs and adults. Investigations on the residual effects of insecticides are important for nymph control due to the long egg-hatching period, and for adult control because FD epidemics are sustained by infectious adults colonizing vineyards from the surrounding areas [21,22]. The results of these trials could help to plan adequate control strategies against *S. titanus* nymphs and adults, complying with the European Union’s achievement of the sustainable use of pesticides (Directive 2009/128/EC). 

## 2. Materials and Methods

### 2.1. Experimental Sites and Insecticides Used in Conventional Viticulture

The efficacy of insecticides used in conventional viticulture was evaluated in two vineyards located in the Veneto region (north-eastern Italy) in the 2022 growing season. The first vineyard (SPF) was located at San Pietro di Feletto (45°54′43″ N, 12°14′12″ E, 214 m a.s.l.) and the second (FM) at Fumane (45°32′34″ N, 10°52′39″ E, 289 m a.s.l.). SPF vineyard was characterized by cultivar Glera, Sylvoz training system, 3.5 m × 1.65 m of planting space. FM vineyard was characterized by cultivar Corvina, Pergola training system, 3.0 m × 0.8 m of planting space. The same protocol and insecticide formulations were used in both vineyards (Table 1). No insecticides other than the comparison ones were applied in the experimental vineyards during the field trials. The experimental plan was a randomized block design with four replicates per treatment represented by plots of about 600 m^2^ (SPF) and about 850 m^2^ (FM). Insecticides were applied on June 10 in SPF and on June 15 in FM vineyards, when the third instar nymphs of *S. titanus* were dominant. Insecticides were sprayed using a farm’s atomizer with pressure 10 bar, driving speed of 6.0 km/h, Albuz core disc nozzles in SPF vineyard, pressure 15 bar, driving speed of 6.5 km/h, MFT core disc nozzles in FM vineyard. The application volume was 10 hL/ha, with the exception of flupyradifurone (8 hL/ha). Samplings were carried out before and seven days after insecticide application by examining 100 basal leaves per replicate (5 leaves per grapevine) for a total of 400 leaves per treatment. In the FM vineyard, sampling occurred also on suckers developed along the grapevine trunk, counting the *S. titanus* nymphs present on 50 suckers per replicate for a total of 200 suckers per treatment.

### 2.2. Experimental Sites and Insecticides Used in Organic Viticulture

The efficacy of insecticides used in organic viticulture was evaluated in two vineyards located in the Veneto region (north-eastern Italy) in the 2022 growing season (Table 1). The first vineyard (SAR) was located at Sarmede (45°58′22″ N, 12°22′45″ E, 103 m a.s.l.) and was characterized by cultivar Glera, Sylvoz training system, 3.5 × 1.65 m of planting space. The second vineyard (STA) was located at Stallavena (45°32′15″ N, 11°00′07″ E, 219 m a.s.l.) and was characterized by cultivar Corvina, Pergola training system, 4.0 × 1.0 m of planting space. The protocol was similar to that previously described for conventional vineyards but in this case, insecticides were applied twice, the first time on 6 June (SAR) or 9 June (STA), when the second instar nymphs were dominant, and the second time on 13 June (SAR) or 15 June (STA). The application volume was 10 hL/ha, using a farm’s atomizer, pressure 8 bar, driving speed of 6.0 km/h, Albuz core disc nozzles in both vineyards. According to producer instructions, the pH of solution was 6.5–6.8 for pyrethrins and pyrethrins + ethoxylated sorbitan monooleate, and 5.5 for azadirachtin. For potassium salts of fatty acids the water hardness was <20 French degrees (°fH). The experimental plan was a randomized block design with four replicates per treatment, represented by plots of about 600 m^2^ (SAR) and about 1700 m^2^ (STA). In the trial performed in the SAR vineyard, samplings before and after insecticide application were done on basal leaves (100 per replicate), while in the STA vineyard, suckers (50 per replicate) were considered for sampling. 

### 2.3. Insecticide Residual Activity in Semi-Field Conditions

Semi-field trials to evaluate insecticide residual activity on *S. titanus* nymphs and adults were carried out in the experimental farms of the University of Padova (Agripolis, Legnaro) and the University of Verona (Villa Lebrecht, San Pietro in Cariano) in the 2022 growing season. These trials were carried out on irrigated one-year potted vines cultivar Chardonnay kept under a shading net. Shoot thinning was performed to obtain at least two grapevine shoots per vine. The effects of 11 products containing active ingredients authorized in conventional or organic viticulture were evaluated (Table 1); a control treated with tap water was included for comparison. In Verona trials, acrinathrin was not tested, and azadirachtin and *B. bassiana* were evaluated only against nymphs. Treatments comprised, respectively, three (Verona) or four (Padova) replicates, each represented by a potted vine. The trials were performed both on nymphs (second and third instars) and on adults of *S. titanus*. Insecticides were applied in mid-June for nymph trials and in July for adult trials. Potted vines were sprayed with a compression sprinkler (8 L volume), spacing them from each other to avoid drift effects. A shoot of each potted vine was inserted three days after treatment into a tulle sleeve (1.0 × 0.6 m) and 10 nymphs or adults per replicate were released into the tulle sleeves, carefully closed around the stem of the shoot to avoid insects escaping. Samplings were carried out three days after caging. At this purpose, grapevine shoots inside the tulle sleeves were cut and carefully inspected, counting dead and alive individuals. A second release of 10 *S. titanus* nymphs or adults was made seven days after insecticide application, confining them on another shoot as above. After three days of confining, dead and alive individuals were counted following the procedure previously described. Leafhoppers used for these trials were collected from organic vineyards located at San Pietro di Feletto (TV) and Stallavena (VR), respectively for trials at the University of Padova and the University of Verona.

### 2.4. Insecticide Residual Activity in Field Conditions

The residual activity of several conventional insecticides was evaluated in a vineyard located at San Pietro di Feletto (45°52′19″ N, 12°15′40″ E, 148 m a.s.l) during the 2021 and 2022 growing seasons (Table 1). This vineyard was characterized by cultivar Glera, Sylvoz training system, 3.5 × 1.65 m of planting space. The occurrence of *S. titanus* was negligible. Four insecticides (acetamiprid, acrinathrin, flupyradifurone and tau-fluvalinate) were tested in both growing seasons, while deltamethrin, etofenprox and sulfoxaflor in 2022 only. An untreated control was included for comparison. Insecticides were applied (using a farm’s atomizer) according to the maximum dose per hectare, as indicated for leafhoppers in the product labels. Each treatment comprised of four replicates represented by three rows 50-80 m long. In both seasons, two trials were carried out, the first devoted to evaluating the insecticide residual activity against nymphs, the second against adults. For nymphs, insecticide applications were performed on 21 June 2021, and on 10 June 2022, while for adults on 2 August 2021, and on 13 July 2022. Three days from insecticide applications, 10 nymphs or adults, collected from nearby vineyards, were confined on a shoot with a tulle sleeve (1 × 0.60 m). A tulle sleeve was installed in a central row of each replicate, for a total of 40 individuals per treatment. Three days after caging, dead and alive individuals were counted, after cutting the shoots and pouring the tulle sleeve contents into a basin for a careful check. The same procedure was performed seven days after insecticide applications. 

### 2.5. Data Analysis

Data obtained from each trial were analyzed using a generalized linear mixed model with the GLIMMIX procedure of SAS^®^ (ver. 9.4; SAS Institute Inc., Cary, NC, USA). In field trials, the number of *S. titanus* per basal leaf or sucker after treatment was used as the dependent variable, both in organic and conventional vineyards, while the type of insecticide was considered as the factor of variation. In the conventional insecticide efficacy trial, the sampling unit and experiment arrangement was identical, thus data from both fields were used to run the model, and the vineyard was considered as a random effect term in the model in order to contribute to the error calculation. In the organic insecticide trial, data from the two fields were analyzed separately due to the different sampling units (i.e., basal leaves in SAR and suckers in STA). In trials aimed at evaluating insecticide residual activity, the number of dead individuals was considered as a dependent variable. The factor of variation (type of insecticide) was tested using an *F-test* (α = 0.05). Comparisons of the mean numbers of *S. titanus* per leaf or sucker in open field trials and dead *S. titanus* in residual activity trials in different treatments were performed using a *t*-test (α = 0.05) on the least-square means. The degrees of freedom were estimated with the Kenward–Roger method, which can calculate non-integer values for error terms. Before the analysis, and data were checked for model assumptions. The model was run on data transformed to log (*n* + 1), while untransformed data are shown in the figures. The effectiveness of the insecticides was calculated according to the Henderson and Tilton formula [35] in field trials and with the Abbott formula (1925) [36] for the residual activity trials. 

## 3. Results

### 3.1. Efficacy of Conventional Insecticides against S. titanus Nymphs in Open Field (Basal Leaves)

**Figure 1:**
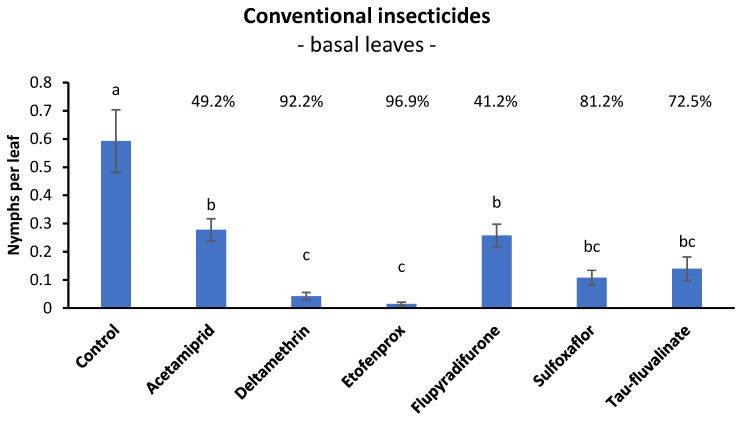
Effects of six insecticides on *S. titanus* nymphs (mean ± SE) evaluated at seven days from their application (SPF and FM vineyard). Different letters indicate significant differences at the *t*-test (α = 0.05). For each insecticide the Henderson and Tilton efficacy (%) is also reported.

No differences among treatments were detected prior to insecticide applications (F = 0.52; d.f. = 6, 48; *p* = 0.788). After insecticide application, the differences among treatments became significant (F = 21.76; d.f. = 6, 48; *p* < 0.0001). All insecticides differed from the control, but deltamethrin and etofenprox were more effective than acetamiprid and flupyradifurone; sulfoxaflor and tau-fluvalinate showed intermediate effects (Figure 1).

Henderson and Tilton efficacy agreed with statistical analysis showing the highest efficacy values for deltamethrin and etofenprox (>90%), the lowest values for flupyradifurone and acetamiprid (<50%) and intermediate values for sulfoxaflor and tau-fluvalinate (Figure 1).

### 3.2. Efficacy of Conventional Insecticides against S. titanus Nymphs in Open Field (Suckers)

**Figure 2:**
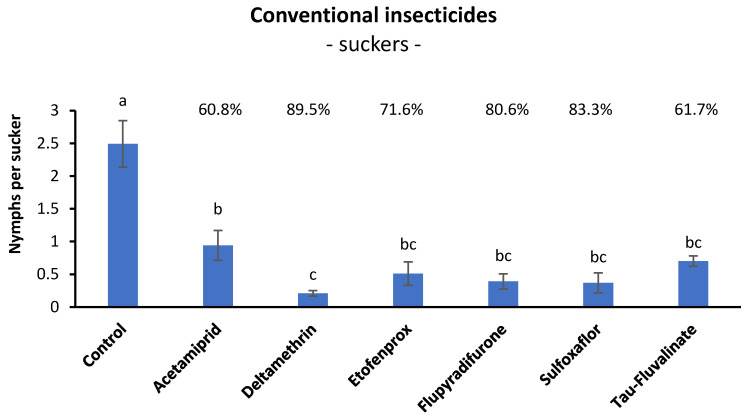
Effects of six insecticides on *S. titanus* nymphs (mean ± SE) evaluated at seven days from their application (FM vineyard). Different letters indicate significant differences at the *t*-test (α = 0.05). For each insecticide the Henderson and Tilton efficacy (%) is also reported.

Before insecticide application there were no differences among treatments (F = 0.58; d.f. = 6, 21; *p* = 0.743), while insecticide application caused significant effects on *S. titanus* nymphs (F = 14.41; d.f. = 6, 21; *p* < 0.0001). All insecticides differed from the control, and among them, deltamethrin was more effective than acetamiprid (Figure 2). The remaining insecticides caused intermediate effects.

Henderson and Tilton efficacy agreed with statistical analysis showing the highest efficacy value for deltamethrin (around 90%), the lowest efficacy value for acetamiprid (around 60%) and intermediate values for the other insecticides (Figure 2).

### 3.3. Efficacy of Organic Insecticides against S. titanus Nymphs in Open Field (Basal Leaves)

**Figure 3:**
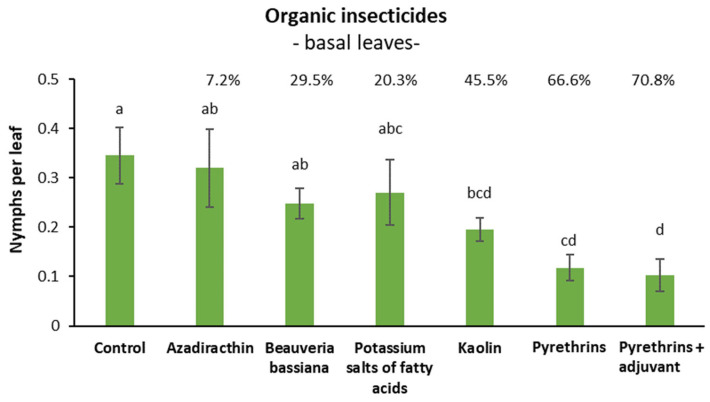
Effects of two applications of five insecticides or kaolin on *S. titanus* nymphs (mean ± SE) evaluated at 14 days from the first application (SAR vineyard). Different letters indicate significant differences at the *t*-test (α = 0.05). For each product the Henderson and Tilton efficacy (%) is also reported.

Before insecticide application, no differences among treatments were found (F = 0.03; d.f. = 6, 21; *p* = 0.999). Later, insecticide application affected *S. titanus* nymph densities (F = 4.65; d.f. = 6, 20.04; *p* = 0.004). Only pyrethrin-based insecticides and kaolin differed significantly from the control without differing each other. Azadirachtin and *B. bassiana* were significantly less effective than the two pyrethrin-based insecticides (Figure 3).

Henderson and Tilton efficacy agreed with statistical analysis showing the highest efficacy values for pyrethrin-based insecticides (around 70%) and the lowest efficacy for azadirachtin (<10%). The remaining products showed intermediate efficacy levels (Figure 3).

### 3.4. Efficacy of Organic Insecticides against S. titanus Nymphs in Open Field (Suckers)

**Figure 4:**
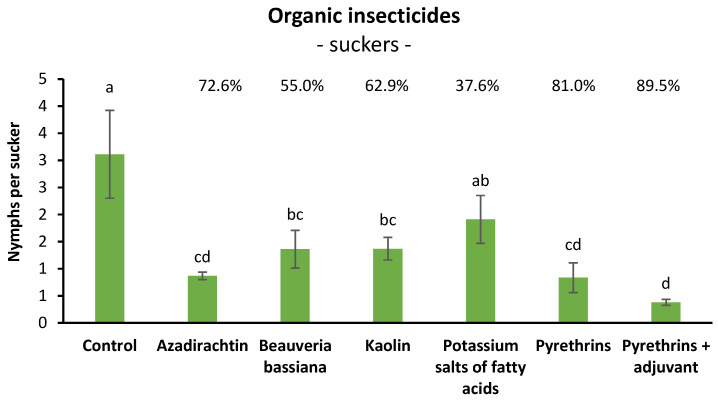
Effects of two applications of five insecticides or kaolin on *S. titanus* nymphs (mean ± SE) evaluated at 14 days from the first application (STA vineyard). Different letters indicate significant differences at the *t*-test (α = 0.05). For each product the Henderson and Tilton efficacy (%) is also reported.

When suckers were sampled prior to insecticide application, there were no differences among treatments (F = 0.42; d.f. = 6, 21; *p* = 0.859). Later, insecticide application caused significant effects on *S. titanus* nymphs (F = 6.61; d.f. = 6, 21; *p* = 0.0005). All products, except potassium salts of fatty acids, differed significantly from the control. The effects of pyrethrins + adjuvant were more impactful than those of *B. bassiana* and kaolin (Figure 4).

Henderson and Tilton efficacy agreed with statistical analysis showing the highest efficacy values for pyrethrin-based insecticides (>80%) and azadirachtin (>70%), and a moderate efficacy for kaolin and *B. bassiana* (>50%) (Figure 4).

### 3.5. Insecticide Residual Activity in Semi-Field Trials

#### 3.5.1. Against *S. titanus* Nymphs

**Figure 5:**
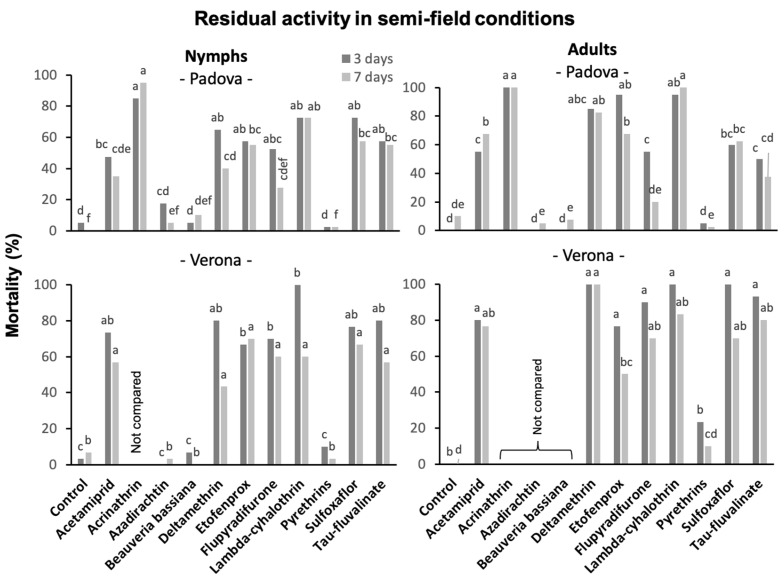
Mortality rates of *S. titanus* nymphs and adults confined on leaves three and seven days after insecticide application in semi-field conditions in Padova and Verona Universities. Different letters above the columns with the same color indicate significant differences at the *t*-test (α = 0.05).

In the trial carried out at the University of Padova, *S. titanus* nymphs were significantly affected by insecticides when confined on shoots of potted vines three and seven days after their application (3 days: F = 22.03; d.f. = 11, 36; *p* < 0.0001; seven days: F = 15.41; d.f. = 11, 36; *p* < 0.0001). At three days, only conventional insecticides determined higher mortality rates compared to the control. Among them, acetamiprid was less effective than acrinathrin, which caused the highest mortality level (>80%) (Figure 5). At seven days, all pyrethroids (acrinathrin, deltamethrin, etofenprox, lambda-cyhalothrin and tau-fluvalinate), acetamiprid and sulfoxaflor differed significantly from the control, and among them the most effective were acrinathrin and lambda-cyhalothrin. 

In the trial carried out at the University of Verona, *S. titanus* nymphs were significantly affected by insecticides when confined on plants three and seven days after their application (three days: F = 44.44; d.f. = 10, 22; *p* < 0.0001; seven days: F = 26.7; d.f. = 10, 22; *p* < 0.0001). At three days, only conventional insecticides determined mortality rates significantly higher than in the control. Etofenprox and flupyradifurone were less effective than lambda-cyhalothrin (Figure 5). At seven days, only conventional insecticides determined mortality rates significantly higher compared to the control and, among them, no significant differences were observed. 

Based on Abbott mortality, the two trials (Padova and Verona Universities) showed the poor efficacy of organic insecticides (i.e., azadirachtin, *B. bassiana* and pyrethrins) (Table S1). In contrast, they stress the efficacy of pyrethroids (i.e., acrinathrin, deltamethrin, etofenprox, lambda-cyhalothrin and tau-fluvalinate) and sulfoxaflor. Acetamiprid and flupyradifurone showed a good efficacy only in the University of Verona trial. Comparing Abbott values at three and seven days, a reduction in efficacy from the first to second caging was observed for deltamethrin and flupyradifurone in the University of Padova trial and for deltamethrin, lambda-cyhalothrin, tau-fluvalinate and acetamiprid in the University of Verona trial. 

#### 3.5.2. Against *S. titanus* Adults

In the trial carried out at the University of Padova, *S. titanus* adults were significantly affected by insecticides when confined on plants three and seven days after their application (three days: F = 30.87; d.f. = 11, 36; *p* < 0.0001; seven days: F = 40.25; d.f. = 11, 36; *p* < 0.0001). At three days, only conventional insecticides determined mortality rates significantly higher than in the control. Among them, acetamiprid, flupyradifurone, tau-fluvalinate and sulfoxaflor were less effective than acrinathrin, which caused a mortality of 100%, and the first three were also less effective than etofenprox and lambda-cyhalothrin (Figure 5). At seven days, flupyradifurone and tau-fluvalinate did not differ anymore from the control; among the other insecticides, acrinathrin and lambda-cyhalothrin were more effective than acetamiprid, etofenprox and sulfoxaflor. 

In the trial carried out at the University of Verona, *S. titanus* adults were significantly affected by insecticides when confined on plants three and seven days after their application (three days: F = 30.22; d.f. = 8, 18; *p* < 0.0001; seven days: F = 16.67; d.f. = 8, 18; *p* < 0.0001). At three days, all insecticides except pyrethrins determined mortality rates significantly higher than in the control without differences among them (Figure 5). At seven days, this result was confirmed for all insecticides except etofenprox, which still differed from the control but was less effective than deltamethrin. 

Based on Abbott mortality, the two trials (Padova and Verona Universities) agreed with the absence of efficacy of pyrethrins and the good efficacy of pyrethroids (i.e., deltamethrin, etofenprox, lambda-cyhalothrin and tau-fluvalinate) and sulfoxaflor. Acrinathrin showed an excellent residual activity in the trial in which it was used. Acetamiprid and flupyradifurone showed a good efficacy only in the University of Verona trial. Comparing Abbott values at three and seven days, a reduction in efficacy from the first to the second release was observed for etofenprox and flupyradifurone in both trials, and for tau-fluvalinate in the University of Padova trial (Table S2). 

### 3.6. Insecticide Residual Activity in Field Trials

#### 3.6.1. Against *S. titanus* Nymphs

**Figure 6:**
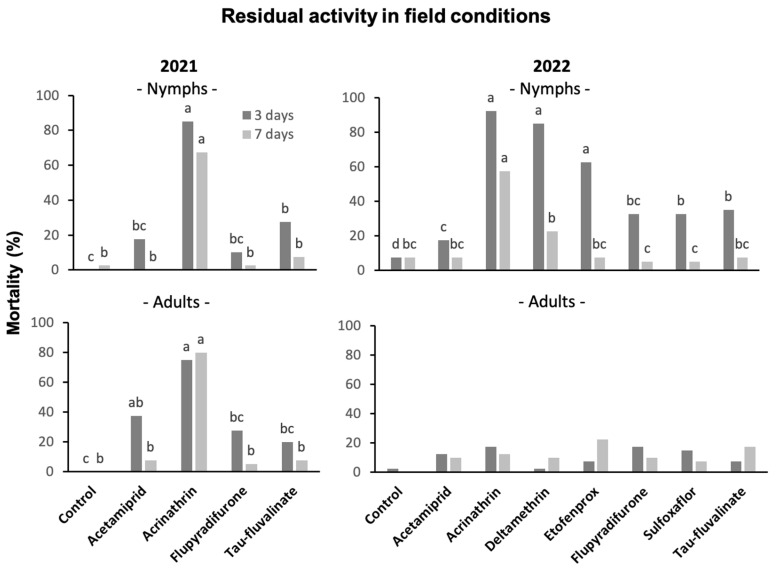
Mortality of *S. titanus* nymphs and adults confined on leaves three and seven days after insecticide application in field conditions in 2021 and 2022. Different letters above the columns with the same color indicate significant differences at the *t*-test (α = 0.05).

In the trial carried out in 2021, insecticides showed significant effects on *S. titanus* nymphs confined on shoots three and seven days from their application (three days: F = 72.89; d.f. = 4, 15; *p* < 0.0001; seven days: F = 12.47; d.f. = 4, 15; *p* < 0.0001). At three days, only acrinathrin and tau-fluvalinate differed from the control, with the first active ingredient significantly more effective than the second (Figure 6). At seven days, only acrinathrin was still significantly different from the control. 

In the trial carried out in 2022, insecticides showed significant effects on *S. titanus* nymphs confined on shoots three and seven days from their application (three days: F =21.53; d.f. = 7, 24; *p* < 0.0001; seven days: F = 5.47; d.f. = 7, 24; *p* < 0.001). At three days all insecticides differed significantly from the control, but the pyrethroids acrinathrin, deltamethrin and etofenprox were more effective than the remaining active ingredients; sulfoxaflor and tau-fluvalinate were more effective than acetamiprid (Figure 6). At seven days, only acrinathrin was still significantly different from the control, as well as from all other insecticides. 

Based on Abbott’s mortality, only acrinathrin showed remarkable residual effects on nymphs both at three (nearly 90%) and seven days (approximately 60%) from insecticide application (Table S3). Deltamethrin and etofenprox had a good efficacy, but only at three days (84% and 60%, respectively). The other active ingredients did not exceed the 30% of efficacy at three days, and showed practically a lack of efficacy at seven days.

#### 3.6.2. Against *S. titanus* Adults

In the trial carried out in 2021, insecticides showed significant effects on *S. titanus* adults confined on shoots three and seven days from their application (three days: F = 10.29; d.f. = 4, 15; *p* < 0.001; seven days: F = 8.09; d.f. = 4, 15; *p* < 0.001). At three days, only acrinathrin and acetamiprid differed from the control without significant differences between them, even if the mortality recorded for the first insecticide was almost double (Figure 6). At seven days, only acrinathrin was still significantly different from the control.

In the trial carried out in 2022, insecticides did not show significant effects on *S. titanus* adults confined on shoots three and seven days from their application (three days: F = 1.27; d.f. = 7, 24; *p* = 0.304; seven days: F = 0.61; d.f. = 7, 24; *p* = 0.740).

Abbott’s mortality on adults was high only for acrinathrin in 2021 trial (around 75% both at three and seven days) (Table S4). In the 2022 trial no insecticides caused a mortality exceeding 20%.

## 4. Discussion

In this study, different approaches were used to evaluate the efficacy of insecticides authorized in Italy for the control of *S. titanus* in conventional and organic vineyards. We carried out field trials against nymphs using a procedure that reflected a winegrowers’ realistic scenario. In these trials, leafhopper nymphs were potentially exposed to insecticides through topical, residual and ingestion (particularly important for systemic insecticides such as neonicotinoids, sulfoximines and butenolides) routes. Semi-field trials were designed to evaluate the mortality of nymphs and adults exposed to aged residues in controlled conditions, to disentangle the effect of residual exposure from the other routes of exposure. Finally, field tests were planned to assess the impact of insecticide residual activity in realistic conditions. 

### 4.1. The Efficacy of Conventional Insecticides against Nymphs in Field Conditions

Among products authorized in conventional viticulture, the most effective insecticides, when *S. titanus* was sampled on basal leaves, were the pyrethroids (IRAC Group 3A) etofenprox (96.9%) and deltamethrin (92.2%). The third pyrethroid, i.e., tau-fluvalinate, was less effective (72.5%) than sulfoxaflor (81.2%, IRAC group 4C). The efficacy of acetamiprid (IRAC group 4A) and flupyradifurone (IRAC group 4D) was much lower (49.2% and 41.2%, respectively). When *S. titanus* was sampled on suckers, a higher efficacy was observed for flupyradifurone and acetamiprid (80.6% and 60.8%, respectively).

The mean efficacy levels of insecticides tested in our trials were compared with values reported in the literature, calculated according to Henderson and Tilton formula. For deltamethrin, our mean efficacy value (90.8%) was lower than those reported by Zidaric et al. (2013) [29] (100% in a single trial) and Colleluori et al. (2020) [37] (98.5% as an average of four trials). Data from the literature on the efficacy of etofenprox [38,39,40] report values (87% as a mean of trials) similar than those found in the present paper (84.3%). The third pyrethroid in this comparison, tau-fluvalinate, was much less effective in the present study (67.1% as a mean of trials) compared to data reported in the literature (Abbott efficacy of 87.4% according to Colleluori et al. (2020) [37]). The most relevant discrepancies between our results and data collected from the literature concerned deltamethrin and tau-fluvalinate, and may be associated with different spraying machines used in trials: backpack sprayers in the literature reports and farm’s atomizers in our trials. Among the remaining insecticides, the most effective resulted sulfoxaflor (mean efficacy of 82.2%). This value is higher than that reported in the literature (60.75% according to Forte et al. (2018) [41]). Unfortunately, this active ingredient was associated with potential negative effects on pollinators, and recently its use in open field conditions has been restricted. The mean efficacy of flupyradifurone was lower (60.9%) than that reported in the literature (77.1%) as a mean of some trials [39,42] where it was sprayed using a backpack sprayer. Acetamiprid efficacy was slightly lower that that reported in the literature (55% vs. 61.7% as a mean of trials carried out by Lavezzaro et al. (2019) [39]). 

In the experimental vineyard where *S. titanus* was sampled also on suckers, the efficacy of pyrethroids slightly decreased compared to that calculated on basal leaves (on average −17%), while that of acetamiprid e flupyradifurone increased (on average +56%). The decrease in efficacy of pyrethroids, acting mainly by contact, could be due to the dilution of the insecticide residues as suckers are rapidly growing shoots (development of new leaves and increase in size of those already present), whereas basal leaves at application timing had already completed their growth. The increase in acetamiprid and flupyradifurone efficacy could be explained by their activity through ingestion, as a consequence of a better absorption of insecticides by younger leaves and a greater acropetal translocation in rapidly growing shoots. This hypothesis should be supported by further experiments, and if demonstrated, sucker management and spraying approaches against this pest could be redefined. 

The higher efficacy of sulfoxaflor compared to acetamiprid and flupyradifurone (all belonging to IRAC Group 4) could be due to the large use of the latter insecticides and the selection pressure exerted upon *S. titanus* populations. This hypothesis could be supported by literature data that reported a higher efficacy of acetamiprid and flupyradifurone in trials conducted some years ago, before the extensive use of these active ingredients. The lower efficacy of these insecticides on basal leaves than on suckers suggests that the limited coverage of basal leaves determines a low-dosage level that is known to be a factor favoring the selection for resistant strains [43,44,45]. This hypothesis should be supported by further experiments, but it is recommended to follow the alternance or rotation measures when planning control strategies against *S. titanus* with these insecticides.

### 4.2. The Efficacy of Organic Insecticides against S. titanus Nymphs in Field Conditions

Among organic insecticides, only pyrethrins and kaolin significantly reduced *S. titanus* population densities. The efficacy of pyrethrins (66.6% on basal leaves and 81% on suckers) was higher than that of kaolin (45.5% on basal leaves and 62.9% on suckers). The efficacy of pyrethrins confirms previous trends [32,39,40]. Results obtained using kaolin (54.2% of efficacy) stress its potential as a complementary tool against several leafhoppers included *S. titanus* [33,46]. Efficacy levels of pyrethrins increased when the adjuvant Mago was added in the trial with sampling on basal leaves (from 66.6% to 70.8%) and, even if only on average, in the trial with sampling on suckers (from 81.0% to 89.5%). Potassium salts of fatty acids did not significantly reduce nymph populations in both trials (20.3% in that sampling on basal leaves and 37.6% in that sampling on suckers), and efficacy values were lower to those reported in the literature (48.1% as a mean of trials carried out by Tacoli et al. (2017) [33] and Forte et al. (2018) [47]). *Beauveria bassiana* significantly reduced nymph population densities in the trial in which suckers were sampled (55.0% of efficacy), but not in the trial in which basal leaves were sampled (29.5%); efficacy values are lower than those reported in the literature (60.3% as a mean of trials carried out by Mori et al. (2014) [32] and Ladurner et al. (2020) [40]) using a backpack sprayer. Contrasting results were obtained with azadirachtin, characterized by a low efficacy (7.2%) in the trial in which basal leaves were sampled and a good efficacy (72.6%) in the trial in which suckers were sampled. The efficacy of azadirachtin against *S. titanus* reported in literature for trials conducted under field conditions is moderate (33.4% as a mean of trials carried out by Bottura et al. (2003) [48] and Mori et al. (2014) [32]). The high efficacy recorded for azadirachtin when sampling was carried out on suckers, could be explained by a greater activity for ingestion as a consequence of a better absorption acropetal translocation in rapidly growing shoots. In fact, in a unique sampling carried out on basal leaves, azadirachtin showed a Henderson and Tilton efficacy of 25.6% (data not reported) much lower than that obtained by sampling suckers. 

### 4.3. Residual Activity of Insecticides in Semi-Field Conditions

In the semi-field trials conducted at Padova University, pyrethroids and sulfoxaflor showed a higher residual activity on both nymphs and adults than acetamiprid and flupyradifurone. In contrast, in trials conducted at Verona University, all conventional insecticides showed a good residual activity up to seven days from insecticide applications. These differences can be attributed to the different origin of *S. titanus* individuals used in the trials. The insects used in Padova trials came from an organically managed vineyard surrounded by conventionally managed vineyards, whereas those used in Verona trials came from an organic vineyard surrounded mainly by woody vegetation. It can be argued that the organic vineyard used as a source of *S. titanus* for Padova trials had been colonized by leafhopper populations subjected to repeated applications of flupyradifurone and acetamiprid in the last years and thus potentially selected for resistance to these active ingredients. Regarding pyrethroids, the best residual activity was recorded for acrinathrin (>80% up seven days release) and lambda-cyhalothrin (on average of the two trials 86% at three-day release and 65% at seven-day release). As expected, organic insecticides (i.e., pyrethrins, azadirachtin and *B. bassiana*) showed poor results in terms of residual activity.

### 4.4. Residual Activity of Insecticides in Field Conditions

Trials conducted in open field in 2021 and 2022 on nymphs showed only for acrinathrin, a remarkable residual activity up to seven days from insecticide application. The efficacy of deltamethrin and etofenprox at three days from insecticide application was good, whereas tau-fluvalinate and the insecticides belonging to IRAC group 4 either showed no residual activity or, as in the case of acetamiprid, it was negligible. Trials conducted on adults confirmed the residual activity of acrinathrin only in the first year, whereas all other active ingredients either showed no efficacy, or did not guarantee sufficient control of *S. titanus* in both years. The lower residual activity of acrinathrin in 2022 compared to 2021 may be due to higher temperatures occurring in 2022. The negative correlation between pyrethroid toxicity against insects and high temperatures is well documented [49,50,51,52,53,54]. Riskallah et al. (1984) [49] demonstrated that permethrin, fenvalerate, deltamethrin, cypermethrin and flucythrinate were more toxic to *Spodoptera littoralis* (Boisd) at 20 °C than at 35 °C. Brown (1987) [50] showed that fenvalerate, flucythrinate and permethrin applied against *Heliothis virescens* (Fabricius) had a lower effectiveness when temperatures increased. Fabellar et al. (1988) [51] demonstrated that cypermethrin and deltamethrin had lower LD50 values at 18 °C than at 33 °C against *Nilaparvata lugens* (Stål) and *Nephotettix* sp. Additionally, recent literature [52,53,54] focused on the changes in the insecticide susceptibility to pyrethroids by *Culex* spp. and *Anopheles* spp., highlighting a deep decrease of their effectiveness at high temperatures (above 30 °C). In our trials, the daily temperatures measured with the closest meteorological station (ARPAV data) to the experimental vineyard were very different between the 2021 and 2022 trials, in particular when experiments were conducted against adults. In the 2021 adult trial, the temperature in the seven days after insecticide application ranged from 16.1 °C to 30.8 °C, with a mean temperature of 23.2 °C while in 2022 the temperature ranged from 20.0 °C to 35.5 °C with a mean temperature of 27.9 °C. Regarding nymph trials, the temperatures in the two years were more similar, ranging from 18.1 °C to 34.4 °C with mean temperature of 26.6° C, and 16.7 °C and 32.3 °C with a mean temperature of 25° C, respectively in 2021 and 2022. In accordance with these considerations, the higher residual efficacies obtained in the semi-field than in field conditions could be due not only to a better coverage by the insecticide solution, but also by the lower temperatures experienced due to the shading net. In fact, leaves exposed to sunlight can have a temperature even 5 degrees higher than shaded ones [55].

## 5. Conclusions

The recent outbreaks of Flavescence dorée are causing extreme concern among winegrowers. They can no longer apply traditional insecticides that proved to be highly effective against *S. titanus* in the past, because of the restrictions by EU authorities. The lower impact of available insecticides has been claimed as a key factor in recent outbreaks of the vector and the related transmitted phytoplasma disease. This situation suggested the need to evaluate the effectiveness of available insecticides. Among conventional insecticides, the most effective were acrinathrin, deltamethrin, lambda-cyhalothrin, etofenprox and sulfoxaflor. However, their residual activity seems to be limited and altered by high temperatures occurring in summer. Moreover, most of them belong to IRAC group 3A, suggesting that resistance could be a problem in the future. Regarding natural products, pyrethrins were the most effective especially when the adjuvant was added. Kaolin proved to be a complementary tool for *S. titanus* management in organic vineyards. Concerning the other organic products that showed a low efficacy on the investigated development stages (L2-L3), further investigations are needed to be re-evaluated against newly hatched individuals (L1), trying to delineate a strategy aimed at decreasing leafhopper densities by integrating insecticides belonging to different IRAC groups. 

The different efficacy showed on basal leaves and on shoots from insecticides acting mainly by contact than those acting through ingestion, suggests canopy or sucker management to concentrate *the S. titanus* individuals on the parts of the canopy most favorable to the insecticide-plant interaction. For pyrethroids and organic insecticides necessary suckering and green pruning should be at least three–four days before spraying, while for neonicotinoids, the shoots may be present but must in any case be sprayed.

The multiple use of pyrethroids and other non-selective insecticides could create issues related to secondary pests. Therefore, monitoring insecticide side-effects is crucial to minimize these risks.

The results of the present study provide precise indications on the strategies to be adopted for the containment of the vector of Flavescence dorée phytoplasma. The two cornerstones of this strategy must be an accurate control of nymphs by using effective insecticides, and the removal of external sources of infectious *S. titanus* adults. In fact, many insecticides have a good knock-down effect against nymphs, and some of them also have a certain level of residual activity. Considering the limited residual activity of most insecticides against adults, the effectiveness of insecticides to control infectious individuals colonizing vineyards from the surrounding areas appears negligible, and therefore attention must be paid to the eradication of abandoned vineyards and American vines growing spontaneously in woody vegetation. 

## Figures and Tables

**Table 1:** Characteristics of insecticides investigated in the different experimental trials.

Management	Formulation	Active Ingredient	Dose (mL or g/hL)	Experimental Trial
Conventional	Decis EVO	Deltamethrin	50	FTE, SRAT *
Conventional	Closer	Sulfoxaflor	40	FTE, SRAT *
Conventional	Epik SL	Acetamiprid	150	FTE, SRAT *
Conventional	Sivanto Prime	Flupyradifurone	60	FTE, SRAT *
Conventional	Mavrik Smart	Tau-fluvalinate	30	FTE, SRAT *
Conventional	Trebon UP	Etofenprox	50	FTE, SRAT *
Conventional	Rufast	Acrinathrin	60	SRAT *
Conventional	Karate Zeon	Lambda-cyhalothrin	25	SRAT *
Organic	Biopiren Plus	Pyrethrins	160	FTE, SRAT *
Organic	Biopiren Plus + Mago	Pyrethrins + ethoxylated sorbitan monooleate	160 + 150	FTE *
Organic	Naturalis	*Beauveria bassiana*	150	FTE, SRAT *
Organic	Flipper	Potassium salts of fatty acids	1500	FTE *
Organic	Neemik TEN	Azadirachtin	390	FTE, SRAT *
Organic	Surround WP	Kaolin	2500	FTE *

* FTE: Field efficacy trial; SRAT: Semi-field residual activity trial.

## Data Availability

The data presented in this study are available from the corresponding author, upon reasonable request.

## Supplementary Materials

The following supporting information can be downloaded at: https://www.mdpi.com/article/10.3390/insects14020101/s1,; Table S1: Results of semi-field trials: Abbott efficacy on *S. titanus* nymphs confined on plants three and seven days after insecticide application.; Table S2: Results of semi-field trials: Abbott efficacy on *S. titanus* adults confined on plants three and seven days after insecticide application.; Table S3: Results of field trials: Abbott efficacy on *S. titanus* nymphs confined on plants three and seven days after insecticide application.; Table S4: Results of field trials: Abbott efficacy on *S. titanus* adults confined on plants three and seven days after insecticide application.
