# Supplementary material for: Efficacy of Conventional and Organic Insecticides against Scaphoideus titanus: Field and Semi-Field Trials
Source: Insects. 2023 Jan 17;14(2):101. doi: 10.3390/insects14020101 (PMC9967193; doi:10.3390/insects14020101)
Supplement: Supplementary file 1 [file insects-14-00101-s001.zip › Table S1.pdf]

insects

Table S1. Results of semi-field trials: Abbott efficacy on *S. titanus* nymphs confined on plants three and seven days after insecticide application.

Active ingredients	Confined three days after insecticide application			Confined seven days after insecticide application		
	Padova	Verona	Average	Padova	Verona	Average
Acetamiprid	44.7%	72.4%	58.6%	35.0%	53.6%	44.3%
Acrinathrin	84.2%			95.0%		
Azadirachtin	13.2%	-3.4%	4.9%	5.0%	-3.6%	0.7%
Beauveria bassiana	0.0%	3.4%	1.7%	10.0%	-7.1%	1.5%
Deltamethrin	63.2%	79.3%	71.3%	40.0%	39.3%	39.7%
Etofenprox	55.3%	65.5%	60.4%	55.0%	67.9%	61.5%
Flupyradifurone	50.0%	69.0%	59.5%	27.5%	57.1%	42.3%
Lambda-cyhalothrin	71.1%	100.0%	85.6%	72.5%	57.1%	64.8%
Pyrethrins	-2.5%	6.9%%	2.2%	2.5%	-3.6%	-0.6%
Sulfoxaflor	71.1%	75.9%	73.5%	57.5%	64.3%	60.9%
Tau-fluvalinate	55.3%	79.3%	67.3%	52.6%	53.6%	53.1%
