# Supplementary material for: Efficacy of Conventional and Organic Insecticides against Scaphoideus titanus: Field and Semi-Field Trials
Source: Insects. 2023 Jan 17;14(2):101. doi: 10.3390/insects14020101 (PMC9967193; doi:10.3390/insects14020101)
Supplement: Supplementary file 1 [file insects-14-00101-s001.zip › Table S2.pdf]

insects

MDPI

Table S2. Results of semi-field trials: Abbott efficacy on *S. titanus* adults confined on plants three and seven days after insecticide application.

Active ingredients	Confined three days after insecticide application			Confined seven days after insecticide application		
	Padova	Verona	Average	Padova	Verona	Average
Acetamiprid	55.0%	80.0%	67.5%	63.9%	76.7%	70.3%
Acrinathrin	100.0%			100.0%		
Azadirachtin	0.0%			-5.6%		
Beauveria bassiana	0.0%			-2.8%		
Deltamethrin	85.0%	100.0%	92.5%	80.6%	100.0%	90.3%
Etofenprox	95.0%	76.7%	85.9%	63.9%	50.0%	57.0%
Flupyradifurone	55.0%	90.0%	72.5%	11.1%	70.0%	40.6%
Lambda-cyhalothrin	95.0%	100.0%	97.5%	100.0%	83.3%	91.7%
Pyrethrins	5.0%	23.3%	14.2%	-8.3%	10.0%	0.9%
Sulfoxaflor	60.0%	100.0%	80.0%	58.3%	70.0%	64.2%
Tau-fluvalinate	50.0%	93.3%	71.7%	30.6%	80.0%	55.3%
