# Supplementary material for: Efficacy of Conventional and Organic Insecticides against Scaphoideus titanus: Field and Semi-Field Trials
Source: Insects. 2023 Jan 17;14(2):101. doi: 10.3390/insects14020101 (PMC9967193; doi:10.3390/insects14020101)
Supplement: Supplementary file 1 [file insects-14-00101-s001.zip › Table S3.pdf]

insects

Table S3. Results of field trials: Abbott efficacy on *S. titanus* nymphs confined on plants three and seven days after insecticide application.

Active ingredients	Confined three days after insecticide application			Confined seven days after insecticide application		
	2021	2022	Average	2021	2022	Average
Acetamiprid	17.5%	10.8%	14.2%	-2,6%	0.0%	0.0%
Acrinathrin	85.0%	91.9%	88.5%	66.7%	54.1%	60.4%
Deltamethrin		83.8%			16.2%	
Etofenprox		59.5%			0.0%	
Flupyradifurone	10.0%	27.0%	18.5%	0%	-2.7%	-1.4%
Sulfoxaflor		27.7%			-2.7%	
Tau-fluvalinate	27.5%	29.7%	28.6%	5.1%	0.0%	2.6%
