# Supplementary material for: Efficacy of Conventional and Organic Insecticides against Scaphoideus titanus: Field and Semi-Field Trials
Source: Insects. 2023 Jan 17;14(2):101. doi: 10.3390/insects14020101 (PMC9967193; doi:10.3390/insects14020101)
Supplement: Supplementary file 1 [file insects-14-00101-s001.zip › Table S4.pdf]

insects

Table S4. Results of field trials: Abbott efficacy on *S. titanus* adults confined on plants three and seven days after insecticide application.

Active ingredients	Confined three days after insecticide application			Confined seven days after insecticide application		
	2021	2022	Average	2021	2022	Average
Acetamiprid	32.4%	10.3%	21.4%	-8.8%	5.3%	-1.8%
Acrinathrin	73.0%	15.4%	44.2%	76.5%	13.2%	44.9%
Deltamethrin		0.0%			5.3%	
Etofenprox		5.1%			15.8%	
Flupyradifurone	21.6%	15.4%	18.5%	11.8%	18.4%	15.1%
Sulfoxaflor		12.8%			5.3%	
Tau-fluvalinate	13.5%	5.1%	9.3%	-8.8%	15.8%	-8.8%
